# Correction: Introducing DDEC6 atomic population analysis: part 1. Charge partitioning theory and methodology

**DOI:** 10.1039/d2ra90050e

**Published:** 2022-05-12

**Authors:** Thomas A. Manz, Nidia Gabaldon Limas

**Affiliations:** Department of Chemical & Materials Engineering, New Mexico State University Las Cruces New Mexico 88003-8001 USA tmanz@nmsu.edu

## Abstract

Correction for ‘Introducing DDEC6 atomic population analysis: part 1. Charge partitioning theory and methodology’ by Thomas A. Manz *et al.*, *RSC Adv.*, 2016, **6**, 47771–47801, https://doi.org/10.1039/C6RA04656H.

The authors regret that there was an error in eqn (25) of the original manuscript. The correct version of eqn (25) is presented below.25
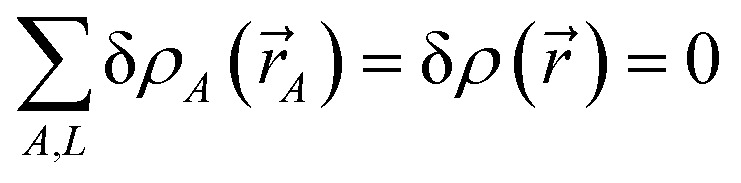


The Royal Society of Chemistry apologises for these errors and any consequent inconvenience to authors and readers.

## Supplementary Material

